# Summary of performance data for technologies to control gaseous, odor, and particulate emissions from livestock operations: Air management practices assessment tool (AMPAT)

**DOI:** 10.1016/j.dib.2016.03.070

**Published:** 2016-04-12

**Authors:** Devin L. Maurer, Jacek A. Koziel, Jay D. Harmon, Steven J. Hoff, Angela M. Rieck-Hinz, Daniel S. Andersen

**Affiliations:** Agricultural and Biosystems Engineering, Iowa State University, Ames, IA 50011, USA

**Keywords:** Air pollution, Mitigation, Livestock production, Odor, Volatile organic compounds, Ammonia, Hydrogen sulfide, Greenhouse gases, Particulate matter, Manure, Literature review

## Abstract

The livestock and poultry production industry, regulatory agencies, and researchers lack a current, science-based guide and data base for evaluation of air quality mitigation technologies. Data collected from science-based review of mitigation technologies using practical, stakeholders-oriented evaluation criteria to identify knowledge gaps/needs and focuses for future research efforts on technologies and areas with the greatest impact potential is presented in the Literature Database tab on the air management practices tool (AMPAT). The AMPAT is web-based (available at www.agronext.iastate.edu/ampat) and provides an objective overview of mitigation practices best suited to address odor, gaseous, and particulate matter (PM) emissions at livestock operations. The data was compiled into Excel spreadsheets from a literature review of 265 papers was performed to (1) evaluate mitigation technologies performance for emissions of odor, volatile organic compounds (VOCs), ammonia (NH_3_), hydrogen sulfide (H_2_S), particulate matter (PM), and greenhouse gases (GHGs) and to (2) inform future research needs.

**Specifications Table**

**Table:** 

Subject area	*Agricultural and Biological Sciences, Engineering, Environmental Sciences*
More specific subject area	*Air Pollution Control, Livestock Production Systems*
Type of data	*Figures, tables*
How data was acquired	*Literature Review of* 265 *articles up to* 2014 [1], [2], [3], [4], [5], [6], [7], [8], [9], [10], [11], [12], [13], [14], [15], [16], [17], [18], [19], [20], [21], [22], [23], [24], [25], [26], [27], [28], [29], [30], [31], [32], [33], [34], [35], [36], [37], [38], [39], [40], [41], [42], [43], [44], [45], [46], [47], [48], [49], [50], [51], [52], [53], [54], [55], [56], [57], [58], [59], [60], [61], [62], [63], [64], [65], [66], [67], [68], [69], [70], [71], [72], [73], [74], [75], [76], [77], [78], [79], [80], [81], [82], [83], [84], [85], [86], [87], [88], [89], [90], [91], [92], [93], [94], [95], [96], [97], [98], [99], [100], [101], [102], [103], [104], [105], [106], [107], [108], [109], [110], [111], [112], [113], [114], [115], [116], [117], [118], [119], [120], [121], [122], [123], [124], [125], [126], [127], [128], [129], [130], [131], [132], [133], [134], [135], [136], [137], [138], [139], [140], [141], [142], [143], [144], [145], [146], [147], [148], [149], [150], [151], [152], [153], [154], [155], [156], [157], [158], [159], [160], [161], [162], [163], [164], [165], [166], [167], [168], [169], [170], [171], [172], [173], [174], [175], [176], [177], [178], [179], [180], [181], [182], [183], [184], [185], [186], [187], [188], [189], [190], [191], [192], [193], [194], [195], [196], [197], [198], [199], [200], [201], [202], [203], [204], [205], [206], [207], [208], [209], [210], [211], [212], [213], [214], [215], [216], [217], [218], [219], [220], [221], [222], [223], [224], [225], [226], [227], [228], [229], [230], [231], [232], [233], [234], [235], [236], [237], [238], [239], [240], [241], [242], [243], [244], [245], [246], [247], [248], [249], [250], [251], [252], [253], [254], [255], [256], [257], [258], [259], [260], [261], [262], [263], [264], [265]
Data format	*Raw*
Experimental factors	*The literature database construction started with compiling literature with the use of online scientific databases, such as Web of Science. Database searches were performed with the keywords: odor, air quality, livestock, poultry, swine, dairy, beef, volatile organic compounds, ammonia, hydrogen sulfide, greenhouse gas, emissions, mitigation, housing, manure storage, and manure land application.*
Experimental features	*The literature review consisted of four steps including (*1*) compilation of literature, (*2*) review of experimental information (reference, experimental design, technology performance, scope of study, etc.), (*3*) compilation and organization of study information into standardized spreadsheets, and (*4*) evaluation of technology and coding for mitigation performance.*
Data source location	*Department of Agricultural and Biosystems Engineering at Iowa State University, Ames, Iowa* 50011*, USA*
Data accessibility	*Data is within this article.*

**Value of the data**

• This data is the most comprehensive performance summary of air pollution control technologies applicable to livestock production systems. This data was collected from 265 published sources [1], [2], [3], [4], [5], [6], [7], [8], [9], [10], [11], [12], [13], [14], [15], [16], [17], [18], [19], [20], [21], [22], [23], [24], [25], [26], [27], [28], [29], [30], [31], [32], [33], [34], [35], [36], [37], [38], [39], [40], [41], [42], [43], [44], [45], [46], [47], [48], [49], [50], [51], [52], [53], [54], [55], [56], [57], [58], [59], [60], [61], [62], [63], [64], [65], [66], [67], [68], [69], [70], [71], [72], [73], [74], [75], [76], [77], [78], [79], [80], [81], [82], [83], [84], [85], [86], [87], [88], [89], [90], [91], [92], [93], [94], [95], [96], [97], [98], [99], [100], [101], [102], [103], [104], [105], [106], [107], [108], [109], [110], [111], [112], [113], [114], [115], [116], [117], [118], [119], [120], [121], [122], [123], [124], [125], [126], [127], [128], [129], [130], [131], [132], [133], [134], [135], [136], [137], [138], [139], [140], [141], [142], [143], [144], [145], [146], [147], [148], [149], [150], [151], [152], [153], [154], [155], [156], [157], [158], [159], [160], [161], [162], [163], [164], [165], [166], [167], [168], [169], [170], [171], [172], [173], [174], [175], [176], [177], [178], [179], [180], [181], [182], [183], [184], [185], [186], [187], [188], [189], [190], [191], [192], [193], [194], [195], [196], [197], [198], [199], [200], [201], [202], [203], [204], [205], [206], [207], [208], [209], [210], [211], [212], [213], [214], [215], [216], [217], [218], [219], [220], [221], [222], [223], [224], [225], [226], [227], [228], [229], [230], [231], [232], [233], [234], [235], [236], [237], [238], [239], [240], [241], [242], [243], [244], [245], [246], [247], [248], [249], [250], [251], [252], [253], [254], [255], [256], [257], [258], [259], [260], [261], [262], [263], [264], [265]

• Researchers and regulatory agencies need a summary and repository of air pollution mitigation technologies data.
• This data can help livestock producers make better decisions on technologies that are available to solve their emissions problems.
• Air pollution mitigation data is grouped by livestock and poultry species, and laboratory, pilot, and farm scale proven performance. This data shows where the knowledge gaps are in regards to emissions mitigation.
• This data shows what tradeoffs may have to be considered in implementing a particular mitigation technology.

## 1 Data

The data presented here is organized reduction values from the literature in regards to livestock emissions mitigation technologies. The data is organized in three Excel files based on the source of emissions: Animal Housing, Land Application and Manure Storage and Handling. Within each file there are four worksheet tabs corresponding to an individual livestock species: Swine, Poultry, Dairy and Beef. Under each species tab there are multiple tables corresponding to a mitigation technology. Within each table there are multiple literature references pertaining to that technology along with the observed reductions in emissions from each reference. Emission reductions in each table correspond to one of six emissions areas: Ammonia, Hydrogen Sulfide, Odor, Dust/Particulates, Volatile Organic Compounds, and Greenhouse Gases.

The data contains 467 technology entries with 670 emissions inputs from 265 papers [1], [2], [3], [4], [5], [6], [7], [8], [9], [10], [11], [12], [13], [14], [15], [16], [17], [18], [19], [20], [21], [22], [23], [24], [25], [26], [27], [28], [29], [30], [31], [32], [33], [34], [35], [36], [37], [38], [39], [40], [41], [42], [43], [44], [45], [46], [47], [48], [49], [50], [51], [52], [53], [54], [55], [56], [57], [58], [59], [60], [61], [62], [63], [64], [65], [66], [67], [68], [69], [70], [71], [72], [73], [74], [75], [76], [77], [78], [79], [80], [81], [82], [83], [84], [85], [86], [87], [88], [89], [90], [91], [92], [93], [94], [95], [96], [97], [98], [99], [100], [101], [102], [103], [104], [105], [106], [107], [108], [109], [110], [111], [112], [113], [114], [115], [116], [117], [118], [119], [120], [121], [122], [123], [124], [125], [126], [127], [128], [129], [130], [131], [132], [133], [134], [135], [136], [137], [138], [139], [140], [141], [142], [143], [144], [145], [146], [147], [148], [149], [150], [151], [152], [153], [154], [155], [156], [157], [158], [159], [160], [161], [162], [163], [164], [165], [166], [167], [168], [169], [170], [171], [172], [173], [174], [175], [176], [177], [178], [179], [180], [181], [182], [183], [184], [185], [186], [187], [188], [189], [190], [191], [192], [193], [194], [195], [196], [197], [198], [199], [200], [201], [202], [203], [204], [205], [206], [207], [208], [209], [210], [211], [212], [213], [214], [215], [216], [217], [218], [219], [220], [221], [222], [223], [224], [225], [226], [227], [228], [229], [230], [231], [232], [233], [234], [235], [236], [237], [238], [239], [240], [241], [242], [243], [244], [245], [246], [247], [248], [249], [250], [251], [252], [253], [254], [255], [256], [257], [258], [259], [260], [261], [262], [263], [264], [265]. Many papers contained data on more than one animal/poultry species, technology and/or an air pollutant emission. Of those 670 emissions inputs, only ~35% of data pertain to farm/field-scale testing. Similarly,~19% of data in the manure storage and handling category,~63% in the manure land application category, and ~43% in the housing category pertain to farm/field-scale. Technologies that were tested at farm/field-scale and had reported emissions reduction > 66% shown in Table 1. These technologies are also highlighted with green color in data (i.e., in three Supplemental Material spreadsheets for Animal Housing, Land Application, and Manure Storage & Handling, respectively). Selected summary of data for the average % reductions in this database is shown in Table 2, Table 3, Table 4, Table 5. Table 1 summarizes technologies that had % reductions >66% for at least one target air pollutant. The following list is a count of specific data categories out of the 467 technology inputs:

• 243 for Swine
• 81 for Poultry
• 86 for Dairy
• 57 for Beef
• 191 for Housing
• 199 for Storage and Handling
• 77 for Land Application

The 670 emission inputs consisted of:

• 207 for Ammonia
• 57 for Hydrogen Sulfide
• 102 for Odor
• 50 for Dust/PM
• 36 for VOCs
• 52 for Carbon Dioxide
• 82 for Methane
• 71 for Nitrous Oxide
• 13 for Carbon Dioxide Equivalents

Complete data can be accessed from public repository: The Air Management Practices Tool (AMPAT) available at Extension and Outreach website, http://www.agronext.iastate.edu/ampat/.

## 2 Experimental design, materials and methods

**Fig. 1:**
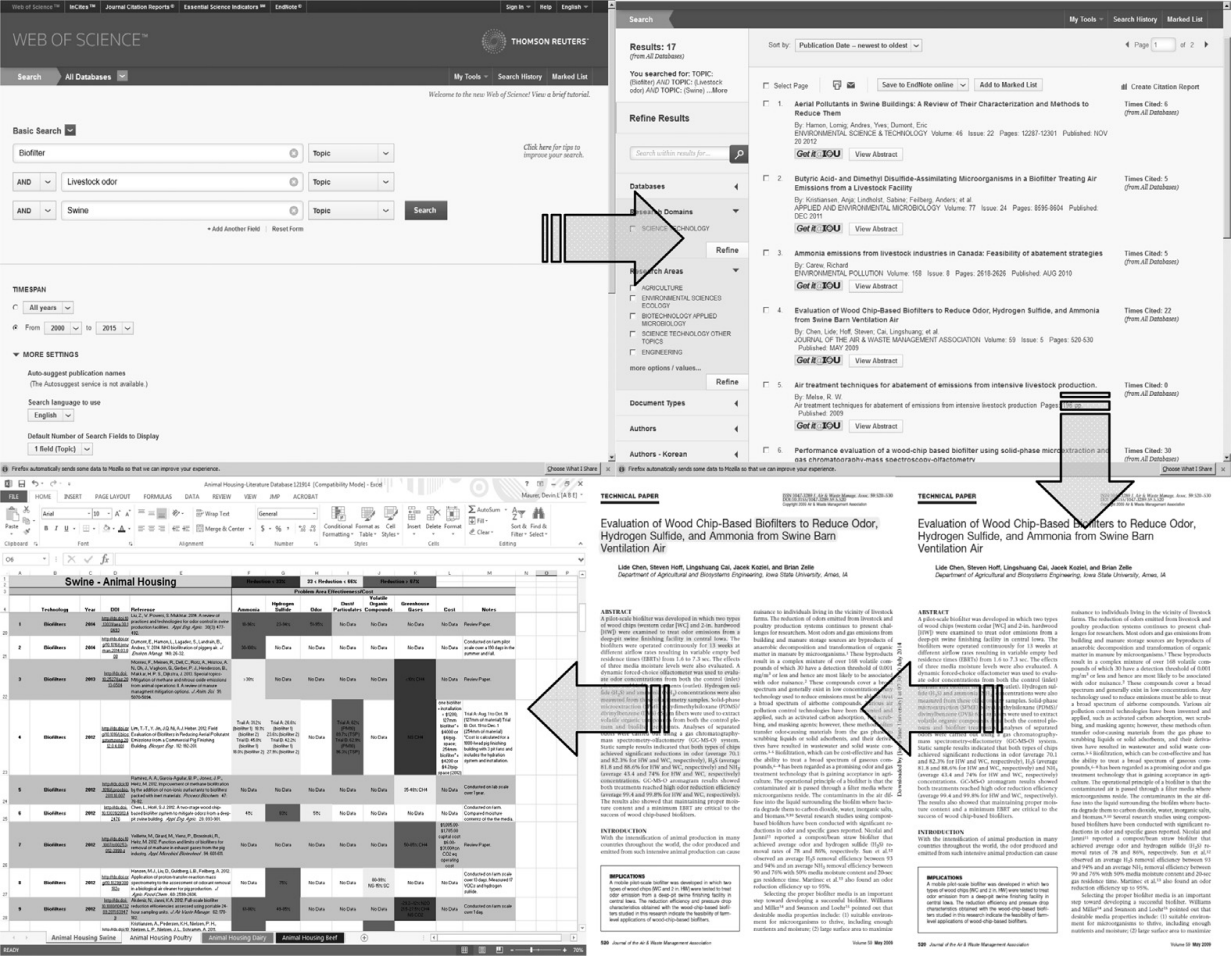
Literature review and information acquisition flow chart.

The literature review consisted of four steps (Fig. 1) including (1) compilation of literature, (2) review of experimental information (reference, experimental design, technology performance, scope of study, etc.), (3) compilation and organization of study information into standardized spreadsheets, and (4) evaluation of technology and coding for mitigation performance. The literature database construction started with compiling literature with the use of online scientific databases, such as Web of Science.

*Database searches were performed with the keywords:*

1. *Odor, air quality, livestock, poultry, swine, dairy, beef, volatile organic compounds, ammonia, hydrogen sulfide, greenhouse gas, emissions, mitigation, housing, manure storage, and manure land application.*

**Fig. 2:**
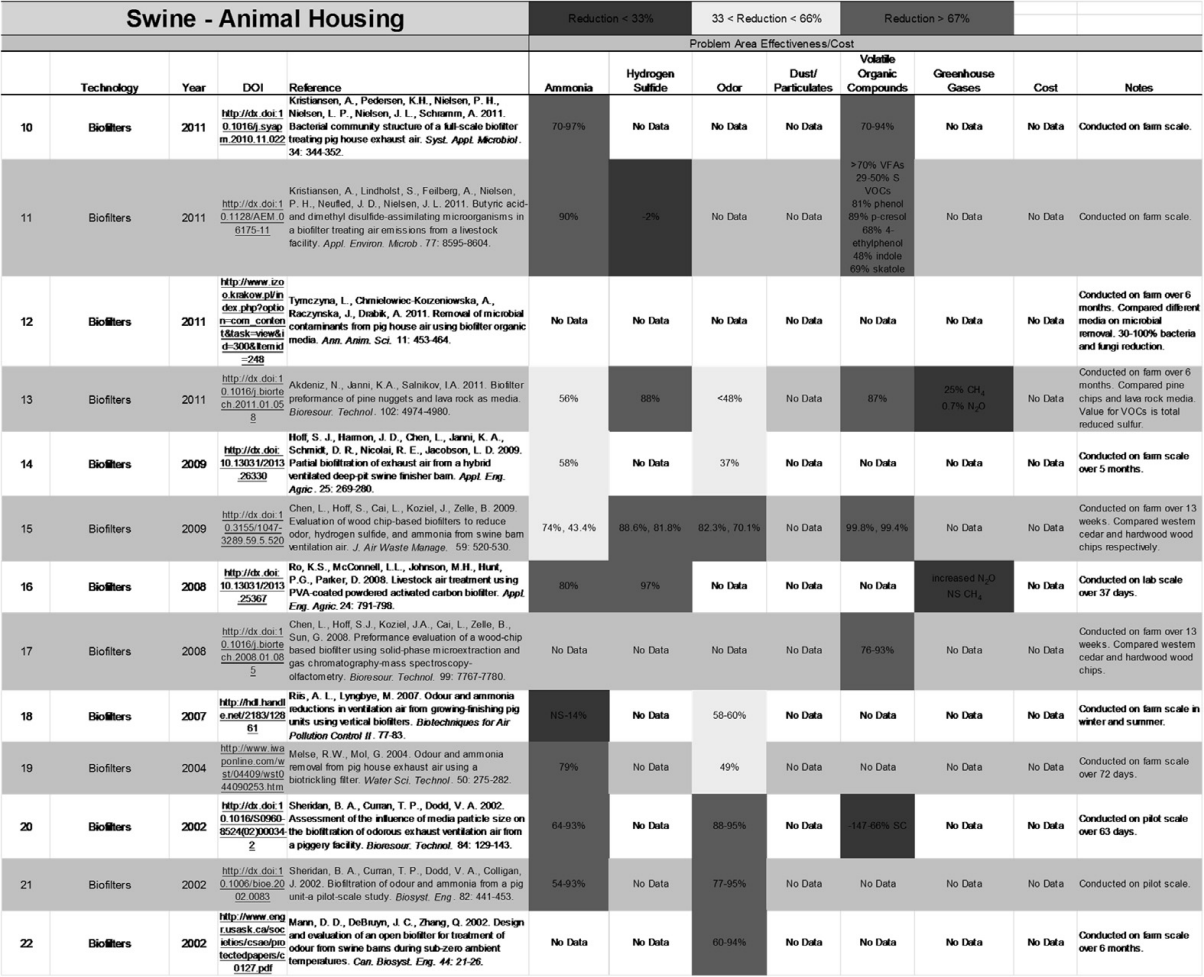
Example of literature database speadsheet. [7], [35], [37], [78], [107], [108], [134], [141], [199], [201], [208], [209], [231] (web link: http://www.agronext.iastate.edu/ampat/database/homepage.html).

The compiled literature was then reviewed and relevant information regarding the experiments conducted, technologies used, emission that were measured, reduction of those emissions, year of publication, DOI or link to literature, cost of implementing the technology, and full reference were extracted. The extracted information was then compiled in standardized spreadsheets according to species and source of emission: housing, manure storage and handling, or manure land application (Fig. 2). If percent emission reductions were not explicedly given in the literature it was calculated if enough other information was avalible using Eq. (1).

(1) $$\%\mathrm{Reduction}=\left(1-\frac{\mathrm{Treated}}{\mathrm{Control}}\right)\times 100$$

*The % reductions for each target emission were color coded in the spreadsheets for quich visual indication of relative effectiveness.*

*The color coding was broken down into three air pollution mitigation technology performance sections:*

1. red=<33% reduction,
2. yellow > 33% and =<67 reduction, or
3. green=>67% reduction.

## Figures and Tables

**Table 1:** Farm/Field Scale-Tested Technologies with Emissions Reductions Greater Than 66%. (See Table 3, Table 4, Table 5 and Supplemental Material for more detailed data).

**Species**	**NH**_**3**_	**H**_**2**_**S**	**Odor**	**PM**	**VOCs**	**CO**_**2**_	**CH**_**4**_	**N**_**2**_**O**	**CO**_**2**_**eq**
**Swine**	scrubbers, urine/feces separation, aeration, solids removal, injection/incorporation and timing	biogas collection/purification	barriers, aeration, impermeable covers, permeable covers	biofilters	biofilter, injection/incorporation	NA	urine/feces separation, aeration, solids removal	solids removal	injection/incorporation
**Poultry**	landscaping	NA	barriers	NA	NA	NA	NA	NA	NA
**Dairy**	NA	biofilters, aeration, impermeable covers	aeration, impermeable covers	NA	aeration, impermeable covers	NA	NA	NA	NA
**Beef**	injection/incorporation	NA	NA	stocking density	NA	NA	NA	manure treatment	NA

*Note:* NA=None available or not performing at this level.

**Table 2:** Swine – selected data summary




*Note:* Only technologies for which emissions reduction >66% were reported for at least one target air pollutant category were included in this table. Values are averages of comparable data across literature in the database. Percent reductions color coded in gray scale by 33% intervals with >66%: White,<−66%: Dark Gray and No Data: Black. Negative values indicate increase in emissions.

**Table 3:** Poultry – selected data summary

*Note:* Only technologies for which emissions reduction > 66% were reported for at least one target air pollutant category were included in this table. Values are averages of comparable data across literature in the database. Percent reductions color coded in gray scale by 33% intervals with > 66%: White,<−66%: Dark Gray and No Data: Black. Negative values indicate increase in emissions.

**Table 4:** Dairy – selected data summary

*Note:* Only technologies for which emissions reduction > 66% were reported for at least one target air pollutant category were included in this table. Values are averages of comparable data across literature in the database. Percent reductions color coded in gray scale by 33% intervals with > 66%: White,<−66%: Dark Gray and No Data: Black. Negative values indicate increase in emissions.

**Table 5:** Beef – selected data summary

*Note:* Only technologies for which emissions reduction > 66% were reported for at least one target air pollutant category were included in this table. Values are averages of comparable data across literature in the database. Percent reductions color coded in gray scale by 33% intervals with > 66%: White,<−66%: Dark Gray and No Data: Black. Negative values indicate increase in emissions.

## References

[1] Aarnink A.J.A., Verstegen M.W.A. (2007). Nutrition, key factor to reduce environmental load from pig production. Livest. Sci..

[2] Aarnink, A.J.A., C.M. Groenestein, N.W.M. Ogink, Aerial pollutants in pig houses; innovative reduction systems in Europe, in: Proceedings of the International Symposium on Animal Environment and Welfare. Chongqing, China, 2013, 90–100.

[3] Aarnink A.J.A., Landman W.J.M., Melse R.W., Zhao Y., Ploegaert J.P., Huyhn T.T.T. (2011). Scrubber capabilities to remove airborne microorganisms and other aerial pollutants from exhaust air of animal houses. Trans. ASABE.

[4] Adrizal A., Patterson P.H., Hulet R.M., Bates R.M., Myers C.A.B., Martin G.P., Thompson J.R. (2008). Vegetative buffers for fan emissions from poultry farms: 2. ammonia, dust, and foliar nitrogen. J. Environ. Sci. Heal..

[5] Adrizal A., Patterson P.H., Hulet R.M., Bates R.M., Despot D.A., Wheeler E.F., Thompson J.R. (2008). The potential for plants to trap emissions from farms with laying hens: 2. ammonia and dust. J. Appl. Poult. Res..

[6] Akdeniz N., Janni K.A. (2012). Full-scale biofilter reduction efficeiencies assessed using portable 24-hour sampling units. J. Air Waste Manag..

[7] Akdeniz N., Janni K.A., Salnikov I.A. (2011). Biofilter performance of pine nuggets and lava rock as media. Bioresour. Technol..

[8] Amon B., Kryvoruchko V., Amon T., Zechmeister-Boltenstern S. (2006). Methane, nitrous oxide and ammonia emissions during storage and after application of dairy cattle slurry and influence of slurry treatment. Agr. Ecosyst. Environ..

[9] Amon M., Dobeic M., Misselbrook T.H., Pain B.F., Phillips V.R., Sneath R.W. (1995). A farm scale study on the use of de-odorase for reducing odor and ammonia emissions from intensive fattening piggeries. Bioresour. Technol..

[10] Applegate T.J., Richert B., Sutton A., Powers W., Angel R. (2008). Diet and Feed Management Practices Affect Air Quality From Poultry and Swine Operations. Purdue Extension AS-582-W.

[11] Arogo J., Westerman P.W., Heber A.J. (2003). A review of ammonia emissions from confined swine feeding operations. Trans. ASAE.

[12] Arogo J., Westerman P.W., Heber A.J., Robarge W.P., Classen J.J. (2006). Ammonia emissions from animal feeding operations. Anim. Agric. Environ..

[13] Battini F., Agostini A., Boulamanti A.K., Giuntoli J., Amaducci S. (2014). Mitigating the environmental impacts of milk production via anaerobic digestion of manure: case study of dairy farm in Po Valley. Sci. Total. Environ..

[14] Baumgartner Environics, Inc. EPI Technology Data, Data & Statistical Analysis Collected and Provided by Murphy-Brown, LLC. 〈http://epiair.com/wp-content/uploads/2012/06/MB-Data-Book.pdf〉 (accessed 1.22.14).

[15] Beauchamp E.G., Kidd G.E., Thurtell G. (1982). Ammonia volatilization from liquid dairy cattle manure in the field. Can. J. Soil. Sci..

[16] J.C. Beddoes, K.S. Bracmort, R.T. Burns, W.F. Lazarus, An analysis of energy production costs from anaerobic digestion systems on U.S. livestock production facilities. USDA NRCS Technical Note. No. 1, 2007.

[17] Bender M.R., Wood C.W. (2007). Above and below ground measurements of greenhouse gases from swine effluent amended soil. Commun. Soil. Sci. Plan..

[18] Berg W., Brunsch R., Pazsiczki I. (2006). Greenhouse gas emissions from covered slurry compared with uncovered during storage. Agric. Ecosyst. Environ..

[19] Bernal M.P., Alburquerque J.A., Moral R. (2009). Composting of animal manures and chemical criteria for compost maturity assessment. A review. Bioresour. Technol..

[20] Bertora C., Alluvione F., Zavattaro L., Willem van Groenigen J., Velthof G., Grignani C. (2008). Pig slurry treatment modifies slurry composition, N_2_O, and CO_2_ emissions after soil incorporation. Soil. Biol. Biochem..

[21] Bhandral R., Bittman S., Kowalenko G., Buckley K., Chantigny M.H., Hunt D.E., Bounaix F., Friesen A. (2009). Enhancing soil infiltration reduces gaseous emissions and improves N uptake from applied dairy slurry. J. Environ. Qual..

[22] Blanes-Vidal V., Hansen M.N., Pedersen S., Rom H.B. (2008). Emissions of ammonia, methane, and nitrous oxide from pig houses and slurry: Effects of rooting material, animal activity and ventilation flow. Agric. Ecosyst. Environ..

[23] R.W. Bottcher, R.D. Munilla, G.R. Baughman, K.M. Keener. Designs for windbreak walls for mitigating dust and odor emissions from tunnel ventilated swine buildings, in: Proceedings of the Swine Housing, Proceedings of the First International Conference. St. Joseph, Mich.: ASAE, 2000, pp. 142–146.

[24] Burton C.H., Sneath R.W., Misselbrook T.H., Pain B.F. (1998). The effect of farm scale aerobic treatment of piggery slurry on odour concentration, intensity, and offensiveness. J. Agr. Eng. Res..

[25] Bush K.J., Heflin K.R., Marek G.W., Bryant T.C., Auvermann B.W. (2014). Increasing stocking density reduces emissions of fugitive dust from cattle feedyards. Appl. Eng. Agric..

[26] Cai L., Koziel J.A., Liang Y., Nguyen A.T., Xin H. (2007). Evaluation of zeolite for control of odorants emissions from simulated poultry manure storage. J. Environ. Qual..

[27] Carter S., Sutton A., Stenglein R. (2012). Diet and feed management to mitigate airborne emissions. Ext. Air Qual. Educ. Agric..

[28] Chadwick D.R., Pain B.F., Brookman S.K.E. (2000). Nitrous oxide and methane emissions following application of animal manures to grassland. J. Environ. Qual..

[29] Chadwick D., Sommer S., Thorman R., Fangueiro D., Cardenas L., Amon B., Misselbrook T. (2011). Manure management: Implications for greenhouse gas emissions. Anim. Feed. Sci. Technol..

[30] Chadwick D.R. (2005). Emissions of ammonia, nitrous oxide and methane from cattle manure heaps: effect of compaction and covering. Atmos. Environ..

[31] Chan A.S.K., Parkin T.B. (2001). Effect of land use on methane flux from soil. J. Environ. Qual..

[32] Chang C., Janzen H.H., Cho C.M. (1998). Nitrous oxide emission from long-term manured soils. Soil. Sci. Soc. Am. J..

[33] Chantigny M.H., Angers D.A., Rochette P., Belanger G., Masse D., Cote D. (2007). Gaseous nitrogen emissions and forage nitrogen uptake on soils fertilized with raw and treated swine manure. J. Environ. Qual..

[34] Chastain J.P. (1999). Air quality and odor control from swine production facilities. Chapter 9 in Confined Animal Manure Managers Certification Program Manual.

[35] Chen L., Hoff S., Cai L., Koziel J., Zelle B. (2009). Evaluation of wood chip-based biofilters to reduce odor, hydrogen sulfide, and ammonia from swine barn ventilation air. J. Air Waste Manag..

[36] Chen L., Hoff S.J. (2012). A two-stage wood chip-based biofilter system to mitigate odors from a deep-pit swine building. Appl. Eng. Agric..

[37] Chen L., Hoff S.J., Koziel J.A., Cai L., Zelle B., Sun G. (2008). Performance evaluation of a wood-chip based biofilter using solid-phase microextraction and gas chromatography-mass spectroscopy-olfactometry. Bioresour. Technol..

[38] Cheng W.-H., Chou M.-S., Tung S.-C. (2011). Gaseous ammonia emission from poultry facilities in Taiwan. Environ. Eng. Sci..

[39] Clanton C.J., Schmidt D.R., Jacobson L.D., Nicolai R.E., Goodrich P.R., Janni K.A. (1999). Swine manure storage covers for odor control. Appl. Eng. Agric..

[40] Clanton C.J., Schmidt D.R., Nicolai R.E., Jacobson L.D., Goodrich P.R., Janni K.A., Bicudo J.R. (2001). Geotextile fabric-straw manure storage covers for odor, hydrogen sulfide, and ammonia control. Appl. Eng. Agric..

[41] Classen J.J., Young J.S., Bottcher R.W., Westerman P.W. (2000). Design and analysis of pilot scale biofiltration system for odorous air. Trans. ASAE.

[42] Clemens J., Trimborn M., Weiland P., Amon B. (2006). Mitigation of greenhouse gas emissions by anaerobic digestion of cattle slurry. Agric. Ecosyst. Environ..

[43] Costa A., Chiarello G.L., Selli E., Guarino M. (2012). Effects of TiO_2_ based photocatalytic paint on concentrations and emissions of pollutants and on animal performance in a swine weaning unit. J. Environ. Manag..

[44] Crosson P., Shalloo L., O’Brien D., Lanigan G.J., Foley P.A., Boland T.M., Kenny D.A. (2011). A review of whole farm systems models of greenhouse gas emissions from beef and dairy cattle production systems. Anim. Feed. Sci. Technol..

[45] Dai X.R., Blanes-Vidal V. (2013). Emissions of ammonia, carbon dioxide, and hydrogen sulfide from swine wastewater during and after acidification treatment: Effect of pH, mixing and aeration. J. Environ. Manag..

[46] De Vries J.W., Aarnink A.J.A., Groot Koerkamp P.W.G., De Boer I.J.M. (2013). Life cycle assessment of segregating fattening pig urine and feces compared to conventional liquid manure management. Environ. Sci. Technol..

[47] Dumont E., Hamon L., Lagadec S., Landrain B., Andres Y. (2014). NH3 biofiltration of piggery air. J. Environ. Manag..

[48] El-Mashad H.M., Zhang R., Arteaga V., Rumsey T., Mitloehner F.M. (2011). Volatile fatty acids and alcohols production during anaerobic storage of dairy manure. Trans. ASABE.

[49] Elwell D.L., Keener H.M., Wiles M.C., Borger D.C., Willett L.B. (2001). Odorous emissions and odor control in composting swine manure/sawdust mixes using continuous and intermittent aeration. Trans. ASAE.

[50] Eriksen J., Adamsen A.P.S., Norgaard J.V., Poulsen H.D., Jensen B.B., Petersen S.O. (2010). Emissions of sulfur-containing odorants, ammonia, and methane from pig slurry: effects of dietary methionine and benzoic acid. J. Environ. Qual..

[51] Eriksen J., Andersen A.J., Poulsen H.V., Adamsen A.P.S., Petersen S.O. (2012). Sulfur turnover and emissions during storage of cattle surry: effects of acidification and sulfur addition. J. Environ. Qual..

[52] Eriksen J., Sorensen P., Elsgaard. L. (2008). The fate of sulfate in acidified pig slurry during storage and following application to cropped soil. J. Environ. Qual..

[53] Fangueiro D., Coutinho J., Chadwick D., Moreira N., Trindade H. (2008). Effect of cattle slurry separation on greenhouse gas and ammonia emissions during storage. J. Environ. Qual..

[54] Fangueiro D., Senbayran M., Trindade H., Chadwick D. (2008). Cattle slurry treatment by screw press separation and chemically enhanced settling: effect on greenhouse gas emissions after land spreading and grass yield. Bioresour. Technol...

[55] Faulhaber C.R., Raman D.R., Burns R.T. (2012). An engineering-economic model for analyzing dairy plug-flow anaerobic digesters: cost structures and policy implications. Trans. ASABE.

[56] Ford S.E., Riskowski. G.L. (2003). Effect of windbreak wall location on ventilation fan performance. Appl. Eng. Agric..

[57] Funk T.L., Mutlu A., Zhang Y., Ellis M. (2004). Synthetic covers for emissions control from earthen embanked swine lagoons part II: negative pressure lagoon cover. Appl. Eng. Agric..

[58] Guarino M., Costa A., Porro M. (2008). Photocatalytic TiO_2_ coating – to reduce ammonia and greenhouse gases concentration and emission from animal husbandries. Bioresour. Technol..

[59] Guo H., Jacobson L.D., Schmidt D.R., Nicolai R.E., Zhu J., Janni K.A. (2005). Development of the OFFSET model for determination of odor-annoyance-free setback distances from animal production sites: part II. Model development and evaluations. Trans. ASAE.

[60] Hamon L., Andres Y., Dumont E. (2012). Aerial pollutants in swine buildings: a review of their characterization and methods to reduce them. Environ. Sci. Technol..

[61] Hanna H.M., Bundy D.S., Lorimor J.C., Mickelson S.K., Melvin S.W., Erbach D.C. (2000). Manure incorporation equipment effects on odor, residue cover, and crop yield. Appl. Eng. Agric..

[62] Hansen M.J., Liu D., Guldberg L.B., Feilberg A. (2012). Application of proton-transfer-reaction mass spectrometry to the assessment of odorant removal in a biological air cleaner for pig production. J. Agric. Food Chem..

[63] Hansen M.N., Henriksen K., Sommer S.G. (2006). Observations of production and emission of greenhouse gases and ammonia during storage of solids separated from pig slurry: effects of covering. Atmos. Environ..

[64] Hansen M.N., Kai P., Moller H.B. (2006). Effects of anaerobic digestion and separation of pig slurry on odor emissions. Appl. Eng. Agric..

[65] Hao X. (2007). Nitrate accumulation and greenhouse gas emissions during compost storage. Nutr. Cycl. Agroecosyst..

[66] Hao X., Chang C., Larney F.J. (2004). Carbon, nitrogen balances and greenhouse gas emission during cattle feedlot manure composting. J. Environ. Qual..

[67] Hao X., Chang C., Larney F.J., Travis G.R. (2001). Greenhouse gas emission during cattle feedlot manure composting. J. Environ. Qual..

[68] J. Harner, R. Maghirang, E. Rozate, Water requirements for controlling dust from open feedlots, in: Proceedings of the Mitigating Air Emissions from Animal Feeding Operations, Des Moines, IA. Iowa State University, 2008, pp. 36-40.

[69] Hartung E., Jungbluth T., Buscher W. (2001). Reduction of ammonia and odor emissions from a piggery with biofilters. Trans. ASAE.

[70] Hartung E., Martinec M., Jungbluth T. (2001). Biofilters-the influence of different filter materials and different operating conditions on the reduction efficiency. Water Sci. Technol..

[71] Hassouna M., Espagnol S., Robin P., Paillat J.-M., Levasseur P., Li Y. (2008). Monitoring NH_3_, N_2_O, CO_2_ and CH_4_ emissions during pig solid manure storage-effect of turning. Compost. Sci. Util..

[72] Heber A.J., Ni J.Q., Lim T.T. (2002). Odor flux measurements at a facultative swine lagoon stratified by surface aeration. Appl. Eng. Agric..

[73] Hernandez G., Trabue S., Sauer T., Pfeiffer R., Tyndall J. (2012). Odor mitigation with tree buffers: Swine production case study. Agric. Ecosyst. Environ..

[74] Hernandez-Ramirez G., Brouder S.M., Smith D.R., Van Scoyoc G.E. (2009). Greenhouse gas fluxes in an eastern corn belt soil: Weather, nitrogen source, and rotation. J. Environ. Qual..

[75] D. Hilborn, J. DeBruyn, Aeration of liquid manure. Ontario Ministry of Agriculture, Food, and Rural Affair Factsheet Order No. 04-033, 2006.

[76] Hjorth M., Nielsen A.M., Nyord T., Hansen M.N., Nissen P., Sommer S.G. (2008). Nutrient value, odour emission and energy production of manure as influenced by anaerobic digestion and separation. Agron. Sustain. Dev..

[77] Hoff J.D., Nelson D.W., Sutton A.L. (1981). Ammonia volatilization from swine manure applied to cropland. J. Environ. Qual..

[78] Hoff S.J., Harmon J.D., Chen L., Janni K.A., Schmidt D.R., Nicolai R.E., Jacobson L.D. (2009). Partial biofiltration of exhaust air from a hybrid ventilated deep-pit swine finisher barn. Appl. Eng. Agric..

[79] Hoff, S.J., D.S. Bundy, J. Harmon, C.D.Johnson, A receptor-based siting strategy for swine production systems, in: Proceedings of the Mitigating Air Emissions from Animal Feeding Operations. Conference Proceedings. Des Moines, IA. Iowa State University, 2008, pp. 15–20.

[80] Hoff S.J., Bundy D.S., Harmon. J.D. (2008). Modeling receptor odor exposure from swine production sources using CAM. Appl. Eng. Agric..

[81] S.J. Hoff, L. Dong, X.W. Li, D.S. Bundy, J.D. Harmon, H. Xin, Odor removal using biomass filters. In: Livestock Environment V, Proceedings of the Fifth International Symposium, St. Joseph, MI.: ASAE, 1997, pp. 101–108.

[82] Honeywell. Ultraviolet Air Treatment Systems. 〈https://customer.honeywell.com/resources/Techlit/TechLitDocuments/50-0000s/50-8887.pdf〉 (accessed 29.04.14).

[83] Hristov A.N., Oh J., Firkins J.L., Dijkstra J., Kebreab E., Waghorn G., Makkar H.P.S., Adesogan A.T., Yang W., Lee C., Gerber P.J., Henderson B., Tricarico J.M. (2013). Mitigation of methane and nitrous oxide emissions from animal operations: I. A review of enteric methane mitigation options. J. Anim. Sci..

[84] Hudson N., Ayoko G.A., Collman G., Gallagher E., Dunlop M., Duperouzel D. (2008). Long-term assessment of efficacy of permeable pond covers for odour reduction. Bioresour. Technol..

[85] Huijsmans J. (2003). Effect of application method, manure characteristics, weather and field conditions on ammonia volatilization from manure applied to arable land. Atmos. Environ..

[86] Huijsmans J., Verwijs B., Rodhe L., Smith K. (2004). Costs of emission-reducing manure application. Bioresour. Technol..

[87] Ikeguchi A., Zhang G., Okushima L., Bennetsen J.C. (2003). Windward windbreak effects on airflow in and around a scale model of a naturally ventilated pig barn. Trans. ASAE.

[88] L.D. Jacobson, B.Pl. Hetchler, D.R. Schmidt, Sampling pit and wall emission for H_2_S, NH_3_, CO_2_, PM & odor from deep-pit finishing facilities, in: Proceedings of the International Symposium on Air Quality and Waste Management for Agriculture (code. 74100). Broomfield, CO.: ASABE, 2007.

[89] L.D. Jacobson, B.Pl. Hetchler, D.R. Schmidt, Reducing H2S, NH3, PM, and odor emissions from deep-pit pig finishing facilities by managing pit ventilation, in: Proceedings of the Mitigating Air Emissions from Animal Feeding Operations. Conference Proceedings, Des Moines, IA. Iowa State University, 2008, pp.41–43.

[90] Jacobson L.D., Guo H., Schmidt D.R., Nicolai R.E., Zhu J., Janni K.A. (2005). Development of the OFFSET model for determination of odor-annoyance-free setback distances from animal production sites: part 1. Review and experiment. Trans. ASAE.

[91] Jarecki M.K., Parkin T.B., Chan A.S.K., Hatfield J.L., Jones R. (2008). Greenhouse gas emissions from two soils receiving nitrogen fertilizer and swine manure slurry. J. Environ. Qual..

[92] Jarret G., Cerisuelo A., Peu P., Martinez J., Dourmad J.-Y. (2012). Impact of pig diets with different fiber contents on the composition of excreta and their gaseous emissions and anaerobic digestion. Agric. Ecosyst. Environ..

[93] Jarret G., Martinez J., Dourmad J.-Y. (2001). Effect of biofuel co-products in pig diets on the excretory patterns of N and C and on the subsequent ammonia and methane emissions from pig effluent. Animal.

[94] S.B. Jerez, S. Mukhtar, W. Faulkner, K.D. Casey, M.S. Borhan, R.A. Smith, Evaluation of electrostatic particulate ionization and BioCurtainTM technologies to reduce air pollutants from broiler houses. ASABE Paper 1110550. St. Joseph, MI, 2011.

[95] T.M. Johnson, B. Murphy, Use of sodium bisulfate to reduce ammonia emissions from poultry and livestock housing, in: Proceedings of the Mitigating Air Emissions from Animal Feeding Operations. Conference Proceedings, Des Moines, IA. Iowa State University, 2008, pp. 74–78.

[96] Johnston N.L., Quarles C.L., Fagerberg D.J., Caveny D.D. (1981). Evaluation of yucca saponin on broiler performance and ammonia suppression. Poul. Sci..

[97] Kai P., Pedersen P., Jensen J.E., Hansen M.N., Sommer S.G. (2008). A whole-farm assessment of the efficacy of slurry acidification in reducing ammonia emissions. Eur. J. Agron..

[98] P. Kai, T. Ellermann, T. Bikkelsen, P. Lofstrom, H. Jorgensen, Effect of stack height on close range dispersion of exhaust air from pig houses, in: International Symposium on Gaseous and Odour Emissions from Animal Production Facilities, Horsens, Denmark, 2003, pp. 264–272.

[99] Kang J., Wang T., Xin H., Wen Z. (2014). A laboratory study of microalgae-based ammonia gas mitigation with potential application for improving air quality in animal production operations. J. Air Waste Manag..

[100] Kaparaju P., Rintala J. (2011). Mitigation of greenhouse gas emissions by adopting anaerobic digestion technology on dairy, sow and pig farms in Finland. Renew. Energy.

[101] D. Karunakaran, Microbial additives to reduce ammonia emission from poultry houses, in: Proceedings of the Mitigating Air Emissions from Animal Feeding Operations. Conference Proceedings, Des Moines, IA. Iowa State University, 2008, pp. 83–84.

[102] Kim K.-Y., Ko H.-J., Kim H.-T., Kim Y.-S., Roh Y.-M., Lee C.-M., Kim C.-N. (2008). Odor reduction rate in confinement pig building by spraying various additives. Bioresour. Technol..

[103] Koger J.B., O׳Brien B.K., Burnette R.P., Kai P., van Kempen M.H.J.G., van Heugten E., van Kempen T.A.T.G. (2014). Manure belts for harvesting urine and feces separately and improving air quality in swine facilities. Livest. Sci..

[104] J.B. Koger, T. van Kempen, G.A. Wossink. Belt manure removal and gasification system to convert dry manure thermally to a combustible gas stream for liquid fuel recovery. Animal and Poultry Waste Management Center. North Carolina State University, Raleigh, North Carolina. 〈http://www.cals.ncsu.edu/waste_mgt/smithfield_projects/recycle/beltgassystem.pdf〉 (accessed 10.08.14).

[105] Koirala K., Ndegwa P.M., Joo H.S., Frear C., Stockle C.O., Harrison J.H. (2013). Impact of anaerobic digestion of liquid dairy manure on ammonia volatilization process. Trans. ASABE.

[106] J. Koziel, X. Yang, T. Cutler, S. Zhang, J. Zimmerman, S. Hoff, W. Jenks, Y. Laor, U. Ravid, R. Armon, H. Van Leeuwen, Mitigation of odor and pathogens from CAFOs with UV/TiO2: Exploring the cost effectiveness. In Proc. Mitigating Air Emissions from Animal Feeding Operations. Conference Proceedings, Des Moines, IA. Iowa State University, 2008, pp.169-173.

[107] Kristiansen A., Lindholst S., Feilberg A., Nielsen P.H., Neufled J.D., Nielsen J.L. (2011). Butyric acid- and dimethyl disulfide-assimilating microorganisms in a biofilter treating air emissions from a livestock facility. Appl. Environ. Microb..

[108] Kristiansen A., Pedersen K.H., Nielsen P.H., Nielsen L.P., Nielsen J.L., Schramm A. (2011). Bacterial community structure of a full-scale biofilter treating pig house exhaust air. Syst. Appl. Microbiol..

[109] I. Lachance, S. Godbout, S.P. Lemay, J.P. Larouche, F. Pouliot, Separation of pig manure under slats: to reduce releases in the environment. ASAE Paper 054159. ASABE, St. Joseph, MI, 2005.

[110] Larney F.J., Hao X. (2007). A review of composting as a management alternative for beef cattle feedlot manure in southern Alberta. Can. Bioresour. Technol..

[111] Le P.D., Aarnink A.J.A., Ogink N.W.M., Becker P.M., Verstegen M.W.A. (2005). Odour from animal production facilities: its relationship to diet. Nutr. Res. Rev..

[112] Li F.B., Li X.Z., Ao C.H., Lee S.C., Hou M.F. (2005). Enhanced photocatalytic degradation of VOCs using Ln3+-TiO2 catalysts for indoor air purification. Chemoshere.

[113] Li H., Xin H., Liang Y., Burns R.T. (2008). Reduction of ammonia emissions from stored laying hen manure through topical application of zeolite, Al+Clear, Ferix-3, or Poultry Litter Treatment. J. Appl. Poult. Res..

[114] Li W., Li Q.-F., Powers W., Karcher D., Angel R., Applegate T.J. (2014). Effects of distillers dried grains with solubles and mineral sources on gaseous emissions. J. Appl. Poult. Res...

[115] Li W., Powers W., Hill G.M. (2011). Feeding distillers dried grains with solubles and organic trace mineral sources to swine and the resulting effect on gaseous emissions. J. Anim. Sci..

[116] Y. Liang, H. Xin, H. Li, R. Gates, E. Wheeler, K. Casey, B. Behrends, D. Burnham, Dietary manipulation to reduce ammonia emission from high-rise layer houses. In Proc. Mitigating Air Emissions from Animal Feeding Operations. Conference Proceedings, Des Moines, IA. Iowa State University, 2008, pp.125-127.

[117] T. Lim, C. Wang, J. Ni, A. Heber, L. Zhao, Effects of aluminum sulfate and aluminum chloride applications to manure on ammonia emission from high-rise layer barn, in: Proceedings of the Mitigating Air Emissions from Animal Feeding Operations. Conference Proceedings. Des Moines, IA. Iowa State University, 2008, pp.85-89.

[118] Lim T.-T., Jin Y., Ni J.Q., Heber A.J. (2012). Field evaluation of biofilters in reducing aerial pollutant emissions from a commercial pig finishing building. Biosyst. Eng..

[119] C.H. Lin, D.D. Walter, H.E. Garrett, R.N. Lerch, Controlling swine odor with windbreaks. In: M. Gold, M.M. Hall (Eds.), Agroforestry Comes of Age: Putting Science into Practice. Paper presented at the Proceedings of the 11th North American Agroforestry Conference, Columbia, Missouri, Columbia, USA: The University of Missouri Center for Agroforestry (UMCA), 2009, pp. 339-348.

[120] Lin H., Wu X., Miller C., Zhu J., Hadlocon L.J., Manuzon R., Zhao L. (2014). Pilot-scale field study for ammonia removal from lagoon biogas using an acid wet scrubber. J. Environ. Sci. Health. Part B.

[121] Lin W.C., Chen Y.P., Tseng C.P. (2013). Pilot-scale chemical-biological system for efficient H_2_S removal from biogas. Bioresour. Technol..

[122] Lin X.-J., Barrington S., Nicell J., Choiniere D., Vezina A. (2006). Influence of windbreaks on livestock odour dispersion plume in the field. Agric. Ecosyst. Environ...

[123] Lin X.-J., Barrington S., Nicell J., Choiniere D., King S. (2007). Livestock odour dispersion as affected by natural windbreaks. Water Air Soil. Pollut..

[124] Liu Z., Powers W. (2014). Greenhouse gases emissions from multi-species animal operations and potential diet effects. Trans. ASABE.

[125] Liu Z., Powers W., Mukhtar. S. (2014). A review of practices and technologies for odor control in swine production facilities. Appl. Eng. Agric..

[126] Lorimor J. (1998). Iowa odor control demonstration project: soil injection.

[127] Loughrin J.H., Cook K.L., Lovanh N. (2012). Recirculating swine waste trough a silicone membrane in an aerobic chamber improves biogas quality and wastewater malodors. Trans. ASABE.

[128] Lovanh N., Warren J., Sistani K. (2010). Determination of ammonia and greenhouse gas emissions from land application of swine slurry: a comparison of three application methods. Bioresour. Technol..

[129] Loyon L., Guiziou F., Beline F., Peu P. (2007). Gaseous emissions (NH_3_, N_2_O, CH_4_ and CO_2_) from the aerobic treatment of piggery slurry-comparison with a conventional storage system. Biosyst. Eng..

[130] Luo J., Klein C.A.M. de, Ledgard S.F., Saggar S. (2010). Management options to reduce nitrous oxide emissions from intensively grazed pastures: a review. Agric. Ecosyst. Environ..

[131] Luo J., Kulasegarampillai M., Bolan N., Donnison A. (2004). Control of gaseous emissions of ammonia and hydrogen sulphide from cow manure by use of natural materials. New Zeal. J. Agr. Res..

[132] Lynch M.B., Sweeney T., Callan J.J., Flynn B., O׳Doherty J.V. (2007). The effect of high and low dietary crude protein and inulin supplementation on nutrient digestibility, nitrogen excretion, intestinal microflora and manure ammonia emissions from finisher pigs. Animal.

[133] G. Malone, G. VanWicklen, S. Collier, Efficiency of vegetative environmental buffers to mitigate emissions from tunnel-ventilated poultry houses, in: Proceedings of the Mitigating Air Emissions from Animal Feeding Operations. Conference Proceedings, Des Moines, IA. Iowa State University, 2008, pp. 27–29.

[134] Mann D.D., DeBruyn J.C., Zhang Q. (2002). Design and evaluation of an open biofilter for treatment of odour from swine barns during sub-zero ambient temperatures. Can. Biosyst. Eng..

[135] R.B. Manuzon, L. Zhao, Laboratory evaluation and modeling of electrostatic precipitation of PM emissions from poultry buildings. ASHRAE Annual Conference, Louisville, KY. Atlanta, GA: American Society Heating, Refrigerating and Air-Conditioning Engineers. T. ASHRAE. vol. 115: part 2, 2009.

[136] Maranon E., Salter A.M., Castrillon L., Heaven S., Fernandez-Nava Y. (2011). Reducing the environmental impact of methane emissions from dairy farms by anaerobic digestion of cattle waste. Waste Manag..

[137] Martinez J., Guiziou F., Peu P., Gueutier V. (2003). Influence of treatment techniques for pig slurry on methane emissions during subsequent storage. Biosyst. Eng..

[138] McCrory D.F., Hobbs P.J. (2001). Additives to reduce ammonia and odor emissions from livestock wastes: a review. J. Environ. Qual..

[139] McGinn S.M., Sommer S.G. (2007). Ammonia emissions from land-applied beef cattle manure. Can. J. Soil. Sci..

[140] Meda B., Hassouna M., Aubert C., Robin P., Dourmad J.Y. (2011). Influence of rearing conditions and manure management practices on ammonia and greenhouse gas emissions from poultry houses. World. Poult. Sci. J..

[141] Melse R.W., Mol G. (2004). Odour and ammonia removal from pig house exhaust air using a biotrickling filter. Water Sci. Technol..

[142] Melse, R.W., N. Ogink and B. Bosma, Multi-pollutant scrubbers for removal of ammonia, odor and particulate matter from animal house exhaust air, in: Proceedings of the Mitigating Air Emissions from Animal Feeding Operations. Conference Proceedings. Des Moines, IA. Iowa State University, 2008, pp.162–168.

[143] Melse R.W., Hofschreuder P., Ogink N.W.M. (2012). Removal of particulate matter (PM10) by air scrubbers at livestock facilities: results of an on-farm monitoring program. Trans. ASABE.

[144] Milllner P.D. (2009). Bioaerosols associated with animal production operations. Bioresour. Technol..

[145] J.R. Miner, S.N. Raja, W. McGregor, Finely ground zeolite as an odour control additive immediately prior to sprinkler application of liquid dairy manure, in: Proceedings of the International Symposium on Ammonia and Odour emissions from Animal Production, Vinkeloord, the Netherlands. Rosmalen, the Netherlands, 1997, pp. 717–720.

[146] Misselbrook T., Nicholson F., Chambers B., Johnson R. (2005). Measuring ammonia emissions from land applied manure: An intercomparison of commonly used samplers and techniques. Environ. Pollut..

[147] Misselbrook T., Prado A. d, Chadwick D. (2013). Opportunities for reducing environmental emissions from forage-base dairy farms. Agr. Food Sci..

[148] Misselbrook T.H., Nicholson F.A., Chambers B.J. (2005). Predicting ammonia losses following the application of livestock manure to land. Bioresour. Technol..

[149] Misselbrook T.H., Smith K.A., Jackson D.R., Gilhespy S.I. (2004). Ammonia emissions from irrigation of dilute pig slurries. Biosyst. Eng..

[150] Monteny G.J., Bannink A., Chadwick D. (2006). Greenhouse gas abatement strategies for animal husbandry. Agric. Ecosyst. Environ..

[151] Montes F., Meinen R., Dell C., Rotz A., Hristov A.N., Oh J., Waghorn G., Gerber P.J., Henderson B., Makkar H.P.S., Dijkstra J. (2013). Special topics- Mitigation of methane and nitrous oxide emissions from animal operations: II. A review of manure management mitigation options. J. Anim. Sci..

[152] P. Moore, Treating poultry litter with aluminum sulfate (Alum). Emissions Management Practice. USDA ARS, 2012 〈http://sera17.ext.vt.edu/〉 (accessed 10.08.14).

[153] B. Mortensen, P. Kai, Odor problems in relation to pig production in Denmark, in: Proceedings of the International Livestock Odor Conference, New Knowledge in Livestock Odor. Ames, IA, 1995, pp. 121–124.

[154] Moset V., Cambra-Lopez M., Estelles F., Torres A.G., Cerisuelo A. (2012). Evolution of chemical composition and gas emissions from aged pig slurry during outdoor storage with and without prior solid separation. Biosyst. Eng..

[155] R. Muhlbauer, J. Puck, B. Puck, R. Burns, A review of manure injection to control odor and ammonia emissions during the land application of manure slurries, in: Proceedings of the Mitigating Air Emissions from Animal Feeding Operations. Conference Proceedings, Des Moines, IA. Iowa State University, 2008, pp. 238–245.

[156] Mukhtar S., Ullman J.L., Carey J.B., Lacey R.E. (2004). A review of literature concerning odors, ammonia, and dust from broiler production facilities: 3. land application, processing, and storage of broiler litter. J. Appl. Poult. Res..

[157] MWPS (1997). Sprinkling Oil to Reduce Dust, Gases, and Odor in Swine Buildings. AED-42. http://www.mwps.org.

[158] MWPS (1983). Swine Housing and Equipment Handbook. http://www.mwps.org.

[159] Nahm K.H. (2007). Feed formulations to reduce N excretion and ammonia emissions from poultry manure. Bioresour. Technol..

[160] R. Nicolai, S. Hoff, Ventilation requirements to prevent pit air up-drafting in a swine finishing barn, in: Proceedings of the Swine Housing II 12–15 October 2003 Conference, Research Triangle Park, NC. ASAE Publication 701P1303. St. Joseph, MI, 2003, pp. 25–30.

[161] Nicolai R., Pohl S., Schmidt D. (2004). Covers for manure storage units. Livestock Development in South Dakota: Environment and Health. FS 925-D.

[162] R.E. Nicolai, B. Hofer, K. Chirpich, Evaluation of a Bio-Curtain. Final Report to the Minnesota Pork Producers, 2008.

[163] R.E. Nicolai, B.J. Hofer, Swine finishing barn dust reduction resulting from an electrostatic space discharge system. ASABE 701P0408, in: Proceedings of the Eighth International Livestock Environment Symposium, St. Joseph, MI.: ASABE, 2008, pp. 125–131.

[164] Nicolai R.E., Janni K.A. (2001). Biofilter media mixture ratio of wood chips and compost treating swine odors. Water Sci. Technol..

[165] O’Neill D.H., Stewart I.W. (1985). State of the Art Report: The Control of Odour Nuisance from Intensive Livestock Buildings.

[166] Osada T., Takada R., Shinzato I. (2011). Potential reduction of greenhouse gas emission from swine manure by using a low-protein diet supplemented with synthetic amino acids. Anim. Feed. Sci. Technol..

[167] Ouellette C., Lemay S., Godbout S., Edeogu I. (2006). Oil application to reduce dust and odour emissions from swine buildings.

[168] Page L.H., Ni J.-Q., Heber A.J., Mosier N.S., Liu X., Joo H.-S., Ndegwa P.M., Harrison J.H. (2014). Characteristics of volatile fatty acids in stored dairy manure before and after anaerobic digestion. Biosyst. Eng..

[169] Paillat J.M., Robin P., Hassouna M., Leterme P. (2005). Predicting ammonia and carbon dioxide emissions from carbon and nitrogen biodegradability during animal waste composting. Atmos. Environ..

[170] Pain B.F., Misselbrook T.H., Nielsen V.P., Voorburg J.H., L׳Hermite P. (1991). Relationships between odour and ammonia emission during and following application of slurries to land. Odour and ammonia emissions from farming.

[171] Pain B.F., Phillips V.R., Clarkson C.R., Misselbrook T.H., Rees Y.J., Farrent J.W. (1990). Odour and ammonia emissions following the spreading of anaerobically treated pig slurry on grassland. Biol. Waste..

[172] Park K.-H., Jeon J.H., Jeon K.H., Kwag J.H., Choi D.Y. (2011). Low greenhouse gas emissions during composting of solid swine manure. Anim. Feed. Sci. Technol..

[173] Parker D.B. (2008). Reduction of odor and VOC emissions from a dairy lagoon. Appl. Eng. Agric..

[174] Parker D.B., Malone G.W., Walter W.D. (2012). Vegetative environmental buffers and exhaust fan deflectors for reducing downwind odor and VOCs from tunnel-ventilated swine barns. Trans. ASABE.

[175] Parker D.B., Gilley J., Woodbury B., Kim K.-H., Galvin G., Bartelt-Hunt S.L., Li X., Snow D.D. (2013). Odorous VOC emissions following land application of swine manure slurry. Atmos. Environ..

[176] Parkin T.B., Kaspar T.C., Singer J.W. (2006). Cover crop effects on the fate of N following soil application of swine manure. Plant Soil..

[177] Parkinson R., Gibbs P., Burchett S., Misselbrook T. (2004). Effect of turning regime and seasonal weather conditions on nitrogen and phosphorus losses during aerobic composting of cattle manure. Bioresour. Technol..

[178] D.A. Paszek, L.D. Jacobson, V.J. Johnson, R.E. Nicolai, Design and management of an oil sprinkling system to control dust, odor, and gases in and from a curtain-sided pig barn. ASAE Paper No. 01-4076. ASAE, St. Joseph, MI, 2001.

[179] Patterson P.H., Adrizal (2005). Management strategies to reduce air emissions: Emphasis-dust and ammonia. J. Appl. Poult. Res..

[180] Pattey E., Trzcinski M.K., Dejardins R.L. (2005). Quantifying the reduction of greenhouse gas emissions as a result of composting dairy and beef cattle manure. Nutr. Cycl. Agroecosys..

[181] Peak Pure Air. How TiO_2_ UV Photocatalytic Oxidation Works 〈http://www.air-oasis-uv-pco-sanitizers.com/how-pco-works.htm〉. (accessed 29.04.14).

[182] Peigne J., Girardin P. (2004). Environmental impacts of farm-scale composting practices. Water Air Soil. Pollut..

[183] Petersen S.O., Amon B., Gattinger A. (2005). Methane oxidation in slurry storage surface crusts. J. Environ. Qual..

[184] Petersen S.O., Andersen A.J., Eriksen J. (2012). Effects of cattle slurry acidification on ammonia and methane evolution during storage. J. Environ. Qual..

[185] Petersen S.O., Dorno N., Lindholst S., Feilberg A., Eriksen J. (2013). Emissions of CH_4_, N_2_O, NH_3_ and odorants from pig slurry during winter and summer storage. Nutr. Cycl. Agroecosyst..

[186] Petersen S.O., Sommer S.G. (2011). Ammonia and nitrous oxide interactions: roles of manure organic matter management. Amin. Feed. Sci. Technol..

[187] Philippe F.X., Cabaraux J.F., Nicks B. (2011). Ammonia emissions from pig houses: influencing factors and mitigation techniques. Agric. Ecosyst. Environ..

[188] Philippe F.X., Nicks B. (2014). Review of greenhouse gas emissions from pig houses: production of carbon dioxide, methane and nitrous oxide by animals manure. Agric. Ecosyst. Environ..

[189] Powers W. (2004). Practices to Reduce Hydrogen sulfide from livestock operations.

[190] W.J. Powers, S.B. Zamzow, B.J. Kerr, Diet modification as a mitigation tool for swine production, in: Proceedings of the International Conference of Agricultural Engineering: Technology for All: Sharing the Knowledge for Development, Brazil. Germany: International Commission of Agricultural Engineering, 2008.

[191] Prapaspongsa T., Christensen P., Schmidt J.H., Thrane M. (2010). LCA of comprehensive pig manure management incorporating integrated technology systems. J. Clean. Prod..

[192] Pratt C., Deslippe J., Tate K.R. (2013). Testing a biofilter cover design to mitigate dairy effluent pond methane emissions. Environ. Sci. Technol..

[193] Pratt C., Walcroft A.S., Tate K.R., Ross D.J., Roy R., Reid M.H., Veiga P.W. (2012). Biofiltration of methane emissions from a dairy farm effluent pond. Agric. Ecosyst. Environ..

[194] Ramirez A.A., Garcia-Aguilar B.P., Jones J.P., Heitz M. (2012). Improvement of methane biofiltration by the addition of non-ionic surfactants to biofilters packed with inert materials. Process. Biochem..

[195] Rao A.G., Gandu B., Swamy Y.V. (2012). Mass transfer dynamics of ammonia in high rate biomethanation of poultry litter leachate. Bioresour. Technol..

[196] L. Reeder, V. Johnson, Using Klasp to reduce poultry housing ammonia emissions, in: Proceedings of the Mitigating Air Emissions from Animal Feeding Operations. Conference Proceedings (). Des Moines, IA. Iowa State University, 2008, pp.79–82.

[197] Regmi S., Onqwandee M., Morrison G., Fitch M. (2007). Effectiveness of porous covers for control of ammonia, reduced sulfur compounds, total hydrocarbons, selected volatile organic compounds, and odor from hog manure storage lagoons. J. Air Waste Manag..

[198] Rigolot C., Espagnol S., Robin P., Hassouna M., Beline F., Paillat J.M., Dourmad J.-Y. (2010). Modelling of manure production by pigs and NH_3_, N_2_O and CH_4_ emissions. Part II: effect of animal housing, manure storage and treatment practices. Animal.

[199] Riis A.L., Lyngbye M. (2007). Odour and ammonia reductions in ventilation air from growing-finishing pig units using vertical biofilters. Biotech. Air Pollut. Control. II.

[200] Ritz, C.W., B.W. Mitchell, B.D. Fairchild, M. Czarick, J.W. Worley, Dust and ammonia control in poultry production facilities using an electrostatic space charge system. In Proc. Mitigating Air Emissions from Animal Feeding Operations. Conference Proceedings (). Des Moines, IA. Iowa State University, 2008, pp.47–50.

[201] Ro K.S., McConnell L.L., Johnson M.H., Hunt P.G., Parker D. (2008). Livestock air treatment using PVA-coated powdered activated carbon biofilter. Appl. Eng. Agric..

[202] Rochette P., Angers D.A., Chantigny M.H., Bertrand N., Côté D. (2004). Carbon dioxide and nitrous oxide emissions following Fall and Spring applications of pig slurry to an agricultural soil. Soil. Sci. Soc. Am. J..

[203] Rochette P., Chantigny M.H., Angers D.A., Bertrand N., Cote D. (2001). Ammonia volatilization and soil nitrogen dynamics following fall application of pig slurry on canola crop residues. Can. J. Soil. Sci..

[204] Rockafellow E.M., Koziel J.A., Jenks W.S. (2012). Laboratory-scale investigation of UV treatment of ammonia for livestock and poultry barn exhaust applications. J. Environ. Qual..

[205] Rodhe L., Pell M., Yamulki S. (2006). Nitrous oxide, methane and ammonia emissions following slurry spreading on grassland. Soil. Use Manag..

[206] Shabtay A., Ravid U., Brosh A., Baybikov R., Eitam H., Laor Y. (2009). Dynamics of offensive gas-phase odorants in fresh and aged feces throughout the development of beef cattle. J. Anim. Sci..

[207] Sharpe R.R., Harper L.A. (2002). Nitrous oxide and ammonia fluxes in a soybean field irrigated with swine effluent. J. Environ. Qual..

[208] Sheridan B.A., Curran T.P., Dodd V.A. (2002). Assessment of the influence of media particle size on the biofiltration of odorous exhaust ventilation air from a piggery facility. Bioresour. Technol..

[209] Sheridan B.A., Curran T.P., Dodd V.A., Colligan J. (2002). Biofiltration of odour and ammonia from a pig unit-a pilot-scale study. Biosyst. Eng..

[210] Sheridan B.A., Hayes E.T., Curran T.P., Dodd V.A. (2004). A dispersion modelling approach to determining the odour impact of intensive pig production units in Ireland. Bioresour. Technol..

[211] Silvaa M.L.B. da, Mezzari M.P., Ibelli A.M.G., Gregory K.B. (2014). Sulfide removal from livestock biogas by azospirillum-like anaerobic phototrophic bacteria consortium. Int. Biodeter. Biodegr..

[212] Sistani K.R., Warren J.G., Lovanh N., Higgins S., Shearer S. (2010). Greenhouse gas emissions from swine effluent applied to soil by different methods. Soil. Sci. Soc. Am. J..

[213] Smith D.R., Owens P.R. (2010). Impact of time to first rainfall event on greenhouse gas emissions following manure applications. Commun. Soil. Sci. Plan..

[214] Smith K.A., Jackson D.R., Misselbrook T.H., Pain B.F., Johnson R.A. (2000). Reduction of ammonia emission by slurry application techniques. J. Agric. Eng. Res..

[215] Sneath R.W. (1988). Centrifugation for separating piggery slurry, 3. Economic effects on aerobic methods of odour control. J. Agric. Eng. Res..

[216] Sommer S.G., Hutchings. N.J. (2001). Ammonia emission from field applied manure and its reduction – invited paper. Euro. J. Agron..

[217] Sommer S.G., Hutchings N. (1995). Techniques and strategies for the reduction of ammonia emission from agriculture. Water Air Soil. Pollut..

[218] Sommer S.G., McGinn S.M., Hao X., Larney F.J. (2004). Techniques for measuring gas emissions from a composting stockpile of cattle manure. Atmos. Environ..

[219] Stackhouse-Lawson K.R., Calvo M.S., Place S.E., Armitage T.L., Pan Y., Zhao Y., Mitloehner F.M. (2013). Growth promoting technologies reduce greenhouse gas, alcohol, and ammonia emissions from feedlot cattle. J. Anim. Sci..

[220] R. Stowell, C. Henry, C. Powers, D. Schulte, Siting animal production facilities and evaluating odor control options using the odor footprint tool, in: Proc. Mitigating Air Emissions from Animal Feeding Operations. Conference Proceedings, Des Moines, IA. Iowa State University, 2008, pp. 2-6.

[221] Sullivan D.G., Wood C.W., Owsley W.F., Norfleet M.L., Wood B.H., Shaw J.N., Adams J.F. (2005). Denitrification following land application of swine waste to Bermuda-grass pasture. Commun. Soil. Sci. Plan..

[222] A. Sutton, Feed Management Practices to Minimize Odors from Swine Operations, 2008.

[223] Sutton A.L., Kephart K.B., Verstegen M.W.A., Canh T.T., Hobbs P.J. (1999). Potential for reduction of odorous compounds in swine manure through diet modification. J. Anim. Sci..

[224] Szogi A.A., Vanotti M.B. (2007). Abatement of ammonia emissions from swine lagoons using polymer-enhanced solid-liquid separation. Appl. Eng. Agric..

[225] Szogi A.A., Vanotti M.B., Stansbery A.E. (2006). Reduction of ammonia emissions from treated anaerobic swine lagoons. Trans. ASABE.

[226] Tenuta M., Mkhabela M., Tremorin D., Coppi L., Phipps G., Flaten D., Ominski K. (2010). Nitrous oxide and methane emission from a coarse-textured grassland soil receiving hog slurry. Agric. Ecosyst. Environ..

[227] Thompson A.G., Wagner-Riddle C., Fleming R. (2004). Emissions of N_2_O and CH_4_ during the composting of liquid swine manure. Environ. Monit. Assess...

[228] Thompson R.B., Meisinger J.J. (2002). Management factors affecting ammonia volatilization from land-applied cattle slurry in the Mid-Atlantic USA. J. Environ. Qual..

[229] Thomsen I.K., Pedersen A.R., Nyord T., Petersen S.O. (2010). Effects of slurry pre-treatment and application technique on short-term N_2_O emissions as determined by a new non-linear approach. Agric. Ecosyst. Environ..

[230] Thorman R.E., Chadwick D.R., Boyles L.O., Matthews R., Sagoo E., Harrison R. (2006). Nitrous oxide emissions during storage of broiler litter and following application to arable land. Int. Congr. Ser..

[231] Tymczyna L., Chmielowiec-Korzeniowska A., Raczynska J., Drabik A. (2011). Removal of microbial contaminants from pig house air using biofilter organic media. Ann. Anim. Sci..

[232] Tyndall J.C., Grala R.K. (2009). Financial feasibility of using shelterbelts for swine odor mitigation. Agroforest. Syst..

[233] J. Tyndall, The use of vegetative environmental buffers for livestock and poultry odor control. In Proc. Mitigating Air Emissions from Animal Feeding Operations. Conference Proceedings. Des Moines, IA. Iowa State University, 2008, pp.21–26.

[234] Tyndall J., Colletti J. (2007). Mitigating swine odor with strategically designed shelterbelt systems: a review. Agroforest. Syst..

[235] Ubeda Y., Lopez-Jimenez P.A., Nicolas J., Calvet S. (2013). Stategies to control odours in livestock facilities: a critical review. Span. J. Agric. Res..

[236] Vaddella V.K., Ndegwa P.M., Joo H.S., Ullman J.L. (2010). Impact of separating dairy cattle excretions on ammonia emissions. J. Environ. Qual..

[237] Vallejo A., García-Torres L., Diez J.A., Arce A., López-Fernández S. (2005). Comparison of N losses (NO_3_-, N_2_O, NO) from surface applied, injected or amended (DCD) pig slurry of an irrigated soil in a Mediterranean climate. Plant Soil..

[238] Van der Stelt B., Temminghoff E.J.M., Van Vliet P.C.J., Van Riemsdijk W.H. (2007). Volatilization of ammonia from manure as affected by manure additives, temperature and mixing. Bioresour. Technol..

[239] VanderZaag A.C., Gordon R.J., Glass V.M., Jamieson R.C. (2008). Floating covers to reduce gas emissions from liquid manure storages: a review. Appl. Eng. Agric...

[240] Vanderzaag A.C., Jayasundara S., Wagner-Riddle C. (2011). Strategies to mitigate nitrous oxide emissions from land applied manure. Anim. Feed. Sci. Technol..

[241] Vandre R., Clemens J. (1997). Studies on the relationship between slurry pH, volatilization processes, and the influence of acidifying additives. Nutr. Cycl. Agroecosyst..

[242] Vanotti M.B., Szogi A.A., Vives C.A. (2008). Greenhouse gas emission reduction and environmental quality improvement from implementation of aerobic waste treatment systems in swine farms. Waste Manag..

[243] Veeken A.H.M., de Wilde V., Willers H.C., Hamelers H.V.M. (2004). Odour abatement in the integrated reactor concept for simultaneous treatment of liquid and sold pig manure fractions. Water Sci. Technol..

[244] Veens T., Namkung H., Leeson S. (2009). Limits to protein in layer diets relative to mitigating ammonia emission. Avian Biol. Res..

[245] Veillette M., Girard M., Viens P., Brzezinski R., Heitz M. (2012). Function and limits of biofilters for removal of methane in exhaust gases from the pig industry. Appl. Microbiol. Biotechnol...

[246] Velthof G.L., Kuikman P.J., Oenema O. (2003). Nitrous oxide emission from animal manures applied to soil under controlled conditions. Biol. Fertil. Soils..

[247] Velthof G.L., Nelemans J.A., Oenema O., Kuikman P.J. (2005). Gaseous nitrogen and carbon losses from pig manure derived from different diets. J. Environ. Qual..

[248] von Bernuth R.D., Hill J.D., Henderson E., Godbout S., Hamel D., Pouliot F. (2005). Efficacy of a liquid/solid isolation system for swine manure. Trans. ASABE.

[249] Wang C., Lu H., Dong D., Deng H., Strong P.J., Wang H., Wu W. (2013). Insight into the effects of biochar on manure composting: evidence supporting the relationship between N_2_O emission and denitrifying community. Environ. Sci. Technol..

[250] Wang K., Huang D., Ying H., Luo H. (2014). Effects of acidification during storage on emissions of methane, ammonia, and hydrogen sulfide from digested pig slurry. Biosyst. Eng..

[251] Wang L., Oviedo-Rondon E.O., Small J., Liu Z., Sheldon B.W., Havenstein G.B., Williams C.M. (2010). Farm-scale evaluation of ozonation for mitigating ammonia concentrations in broiler houses. J. Air Waste Manag..

[252] Wang Y., Dong H., Zhu Z., Liu C., Xin H. (2014). Comparison of air emissions from raw liquid pig manure and biogas digester effluent storages. Trans. ASABE.

[253] Webb J., Pain B., Bittman S., Morgan J. (2010). The impacts of manure application methods on emissions of ammonia, nitrous oxide on crop response – a review. Agric. Ecosyst. Environ..

[254] Webster A.B., Thompson S.A., Hinkle N.C., Merka W.C. (2006). In-house composting of layer manure in high-rise, tunnel-ventilated commercial layer house during an egg production cycle. J. Appl. Poult. Res..

[255] Weiske A., Vabitsch A., Olesen J.E., Schelde K., Michel J., Friedrich R., Kaltschmitt M. (2006). Mitigation of greenhouse gas emissions in European conventional and organic dairy farming. Agric. Ecosyst. Environ..

[256] Whalen S.C. (2000). Nitrogen oxide emission from agricultural soil fertilized with liquid swine waste or constituents. Soil. Sci. Soc. Am. J..

[257] Whitehead T.R., Spence C., Cotta M.A. (2013). Inhibition of hydrogen sulfide, methane, and total gas production and sulfate-reducing bacteria in in vitro swine manure by tannins, with focus on condensed quebracho tannins. Appl. Microbiol. Biotechnol..

[258] A. Winkel, J. Mosquera, N.W.M. Ogink, Removal efficiency of a wire-to-plate electrostatic precipitator for abatement of particulate matter emission from poultry houses. Paper ILES12-0405.Ninth International Livestock Environment Symposium. St. Joseph, MI.: ASABE, 2012.

[259] Winkel A., Mosquera J., Huisin’t Veld J.W.H., Ogink N.W.M., Aarnick A.J.A. (2011). Measures to reduce fine dust emission from poultry: validation of an ionization system on broiler farms.

[260] Yamulki S. (2006). Effect of straw addition on nitrous oxide and methane emissions from stored farmyard manures. Agric. Ecosyst. Environ..

[261] Zhang R., McGarvey J.A., Ma Y., Mitloehner F.M. (2008). Effects of anaerobic digestion and aerobic treatment on the reduction of gaseous emissions from dairy manure storages. Int. J. Agric. Biol. Eng..

[262] Zhang R.H., Westerman P.W. (1997). Solid-liquid separation of animal manure for odor control and nutrient management. Appl. Eng. Agric..

[263] Zhang S.J., Zhu J., Park K.J. (2004). Effects of duration and intensity of aeration on solids decomposition in pig slurry for odour control. Biosyst. Eng..

[264] Zhang Y., Tanaka A., Barber E.M., Feddes J.J.R. (1996). Effects of frequency and quantity of sprinkling canola oil on dust reduction in swine buildings. Trans. ASAE.

[265] Zhang Z.J., Zhu J. (2005). Effectiveness of short-term aeration in treating swine finishing manure to reduce odour generation potential. Agric. Ecosyst. Environ..

